# Correlation between *mazEF* Toxin-Antitoxin System Expression and Methicillin Susceptibility in *Staphylococcus aureus* and Its Relation to Biofilm-Formation

**DOI:** 10.3390/microorganisms9112274

**Published:** 2021-10-31

**Authors:** Aya Abd El rahman, Yasmine El kholy, Rania Y. Shash

**Affiliations:** Department of Medical Microbiology and Immunology, Faculty of Medicine, Cairo University, Cairo 11865, Egypt; yasminelkholy@kasralainy.edu.eg (Y.E.k.); raniayahiashash@cu.edu.eg (R.Y.S.)

**Keywords:** MRSA, MSSA, toxin-antitoxin systems, *mazEF*, biofilm

## Abstract

Methicillin resistance in *Staphylococcus aureus* has become prevalent globally. Moreover, biofilm-formation makes it more difficult to eradicate bacteria by antibiotics. The *mazEF* toxin-antitoxin system encodes for mazF, which acts as an endoribonuclease that cleaves cellular mRNAs at specific sequence motifs (ACA), and mazE, which opposes the mazF action. Our goal was to detect *mazEF* expression in methicillin-resistant *S. aureus* (MRSA) isolates compared with methicillin-sensitive *S. aureus* (MSSA) isolates and determine its relation to methicillin susceptibility as well as biofilm-formation. According to their susceptibility to cefoxitin disks, 100 *S. aureus* isolates obtained from patients admitted to Cairo University Hospitals were categorized into 50 MSSA and 50 MRSA according to their susceptibility to cefoxitin disks (30 µg). Antimicrobial susceptibility and biofilm-formation were investigated using the disk diffusion method and tissue culture plate method, respectively. Finally, using real-time PCR, *mazEF* expression was estimated and correlated to methicillin susceptibility and biofilm formation. Both MRSA and MSSA isolates showed the best sensitivity results with linezolid and gentamicin, where about 88% of MRSA isolates and 96% of MSSA isolates were sensitive to linezolid while 76% of MRSA isolates and 84% of MSSA isolates were sensitive to gentamicin. MRSA isolates were significantly more able to form biofilm than MSSA isolates (*p*-value = 0.037). The *mazEF* expression was significantly correlated to methicillin resistance in *S. aureus* (*p*-value < 0.001), but not to biofilm-formation.

## 1. Introduction

*S. aureus* leads to many community and nosocomial-acquired infections [1]. It results in various diseases, either localized skin infections or fatal systemic diseases [1].

The staphylococcal *mec* operon encodes for a different penicillin-binding protein called (PBP2a) which exhibits less methicillin binding affinity. This makes staphylococci unsusceptible to all β-lactam antibiotics. Besides the *mec* operon, staphylococcal chromosomal cassettes contain genes coding for resistance to antibiotics other than β-lactams [2]. Resistance towards many other antibiotics such as aminoglycosides, quinolones, macrolides-lincosamides-streptogramins, vancomycin, and linezolid has also been shown in *S. aureus* [2].

Biofilm is a polymeric glycocalyx that causes bacterial adherence and allows bacterial spread to other sites. Biofilm makes it more difficult for antimicrobial agents to eliminate infections [3]. In *S. aureus*, biofilm-formation is under control of a quorum sensor called *agr.* The *icaADBC* operon codes for the primary polysaccharide in the biofilm called polysaccharide intercellular adhesion [4]. The low metabolically active cells present deep in the biofilm are more resistant to antibiotics [3].

A toxin-antitoxin (TA) system involves a toxin that interferes with bacterial cell survival and an antitoxin which protects the bacterial cell from the toxin. Toxins are more resistant to stressful conditions than antitoxins. Those systems are commonly expressed in bacterial strains [5]. Regarding TA systems encoded on plasmids, when TA genes are not inherited to daughter cells, the antitoxin is degraded, and the toxin remnants kill those progeny cells in what is called post-segregational killing (PSK) [6]. There are seven well-known TA systems. The classification depends on the technique by which the antitoxin converses the lethal toxic effect, and either the antitoxin is a protein or RNA [5,7]. The first described chromosomal TA system was the *mazEF* system that is classified as type II TA system [8]. The mazF is the toxin with an endoribonuclease activity against cellular mRNAs at a specific sequence (ACA), and the mazE is the antitoxin protein [8]. *S. aureus* genome includes many TA systems such as *mazEF*, *yefM-yoeB*, *omega-epsilon-zetasustem*, *sprA1-sprA1_AS,_* and *sprFG* [9]. Because of the similarity between TA targets and antibiotics targets and the ability of TA systems to cause programmed cell death, TA systems might be used as a target in the treatment of infectious diseases rather than using antibiotics [10].

## 2. Materials and Methods

This study was designated as a descriptive cross-sectional study. It involved approximately 50 MRSA and 50 MSSA isolates collected from clinical specimens from July 2019 to September 2019 at Cairo University Hospitals, Clinical Laboratories, Cairo, Egypt.

The Research Ethics Committee of the Institutional Review Board approved the research (code: M D-37-2019), Faculty of Medicine, Cairo University, Egypt. The study involved bacterial isolates obtained from our University hospital isolates repertoire, so the ethics committee waived the requirement for informed consent.

Identification of bacterial isolates was accomplished by Gram-stained smears and conventional biochemical reactions, including catalase and tube coagulase tests.

### 2.1. Testing for Methicillin Sensitivity

Methicillin sensitivity was screened by the Kirby–Bauer disk diffusion method using a cefoxitin disk (Fox) (30 µg) (Oxoid, Altrincham, UK) and analyzed in consonance with Clinical and Laboratory Standard Institute (CLSI) breakpoints [11]. The diameter of the inhibitory zone was estimated in millimeters. Susceptible isolates have a zone diameter equal to or less than the susceptible breakpoint. It was inhibited by the antimicrobial agent at the achievable concentrations when the recommended dosage for treatment at the site of infection was utilized, indicating potential clinical efficacy; otherwise, the isolate is regarded as resistant [11].

### 2.2. Antimicrobial Susceptibility Testing for MRSA and MSSA Isolates Using Kirby-Bauer Disk Diffusion Method

The antimicrobial agents tested included penicillin (P) (10 units), gentamicin (GEN) (10 µg), tetracycline (TE) (30 µg), levofloxacin (LE) (5 µg), cotrimoxazole (COT) (25 µg), erythromycin (E) (15 µg), clindamycin (CD) (2 µg) and linezolid (LZ) (30 µg) (Himedia, India). A D test was used to detect inducible clindamycin resistance. The sensitivities were analyzed based on CLSI breakpoints [11].

### 2.3. Screening for Biofilm-Formation

The tissue culture plate method (TCP) was used for detecting biofilm-formation [12]. Fresh colonies were inoculated in 2 mL of tryptone soy broth (Oxoid, UK), to which 1% glucose was added; bacterial suspensions were prepared and equalized to 0.5 McFarland standard. The positive and negative controls were represented using *S. aureus* (ATCC-25923) (Cairo University Hospitals, Clinical Laboratories, Cairo, Egypt.) and sterile broth, respectively. Each isolate was represented in three successive wells of the sterile 96-well flat-bottom polystyrene tissue culture plates (Falcon, Becton Dickinson, Franklin Lakes, NJ, USA) and incubated overnight at 37 °C. Then, about 250 µL of phosphate buffer saline (pH 7.2) (Oxford, India) was added to each well twice for washing. Next, about 200 µL of methanol (Biochem, Cairo, Egypt) was added for fixation, and the plates were left at room temperature for 15 min. Then, staining was performed for 5 min using 200 µL of 1% crystal violet, followed by adding sterile distilled water for washing. The plates were left at room temperature until dry. Next, about 200 µL of 96% ethyl alcohol was added to each well. Finally, by utilizing a micro-ELISA auto reader Stat Fax-2100 (Awareness Technology, Palm city, FL, USA), the optical density (OD) of the biofilm was measured at wavelength 492 nm [12]. OD values for each isolate were averaged; OD < 0.12 is considered non-biofilm-forming, OD within 0.12 to 0.2 is weak biofilm-forming, OD within 0.2 to 0.4 is moderate biofilm-forming, and OD > 0.4 is strong biofilm-forming [13].

### 2.4. Expression of mazEF Toxin-Antitoxin Genes in MSSA and MRSA

After bacterial culturing for 24 h, about 1.5 mL (up to 1 × 10^9^ cells measured by spectrophotometer with OD 0.5 to 1 at wavelength 600 nm) was collected in 1.5 mL microcentrifuge tubes. The tubes were centrifuged for 2 min at ≥12,000× *g* rpm. The supernatant was carefully removed, leaving the pellet as dry as possible. The pellet was re-suspended in 100 μL of freshly prepared TE buffer (Thermofisher, Waltham, MA, USA) (10 mM Tris HCl, pH 8.0, 1 mM EDTA) supplemented with lysozyme and incubated for 5 min at 15–25 °C. Then, RNA extraction steps were continued according to the GeneJet RNA purification kit (Thermo Scientific, Vilnius, Lithuania). The extracted total RNA was utilized for cDNA conversion by the QuantiTect reverse transcription kit (Qiagen, Germantown, MD, USA). Extracted RNA, gDNA Wipeout Buffer, and RNase-free water were incubated and mixed at 42 °C for 2 min to eliminate genomic DNA contamination from starting RNA samples effectively. Quantiscript Reverse Transcriptase, Quantiscript RT Buffer, and RT Primer Mix were incubated and added at 42 °C for 15 min to synthesize cDNA. Then the reaction was incubated at 95 °C for 3 min to inactivate Quantiscript Reverse Transcriptase.

Finally, a quantitative RT-PCR was conducted by SYBR Green I (Qiagen, Germantown, MD, USA) on the basis of kit instructions. Specific primers for the target gene *mazEF* (forward primer: ATCATCGGATAAGTACGTCAGTTT, reverse primer: AGAAGGATATTCACAAATGGCTGA) and the housekeeping gene *16S rRNA* (forward primer: GTAGGTGGCAAGCGTTATCC, reverse primer: CGCACATCAGCGTCAG) were used [14,15]. The master mix contained 1 μL of the forward primer, about 1 μL of the reversed primer, 10 μL of SYBR green master mix, 3 μL of cDNA template, and 10 μL of RNAse free water in a total volume of 25 µL. An Applied Biosystems StepOne Thermal Cycler with software version 3.1 (Applied Biosystems, Waltham, MA, USA) was utilized for amplification and analysis in 45 successive cycles. The housekeeping *16S rRNA* gene expression was utilized to normalize the expression levels of the target gene then the analysis was accomplished using the comparative 2^−ΔΔCT^ method [16].

### 2.5. Statistics

Data were analyzed by the Statistical Package for the Social Sciences (SPSS) version 26 (IBM Corp., Armonk, NY, USA). Quantitative data were represented by mean, standard deviation, minimum, maximum, and median, while count, and percentage represented qualitative data. It was found that *mazEF* expression values were highly variable and not normally distributed; therefore, we depended on the median and the inter-quartile range, not the mean, and utilized a non-parametric method (Independent-Samples Mann–Whitney U Test). Considering qualitative data, the Pearson chi-square test was conducted. Those *p*-values less than 0.05 were regarded as statistically significant [17].

## 3. Results

### 3.1. Antimicrobial Susceptibility Pattern

Among all the tested antibiotics, MRSA and MSSA isolates showed the best sensitivity results with linezolid and gentamicin. Within MRSA isolates, about 88% and 76% of the tested isolates were sensitive to linezolid and gentamicin, respectively, whereas, within MSSA isolates, about 96% and 84% of the tested isolates were sensitive to both agents, respectively.

**Figure 1:**
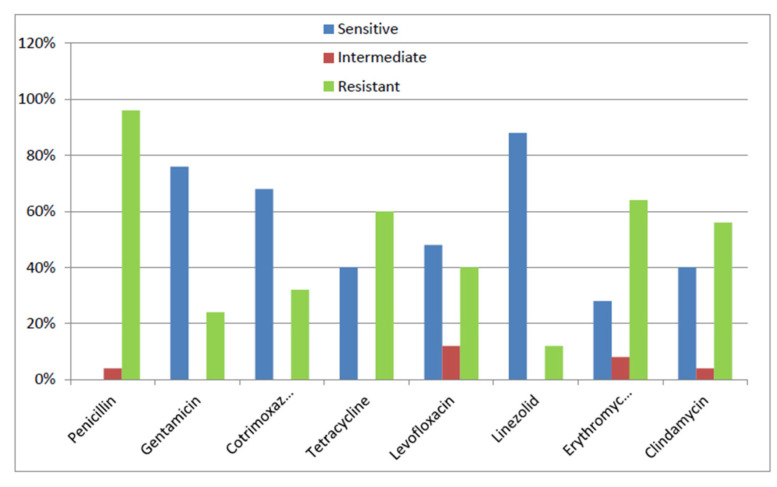
Antimicrobial susceptibility pattern of MRSA isolates.

**Figure 2:**
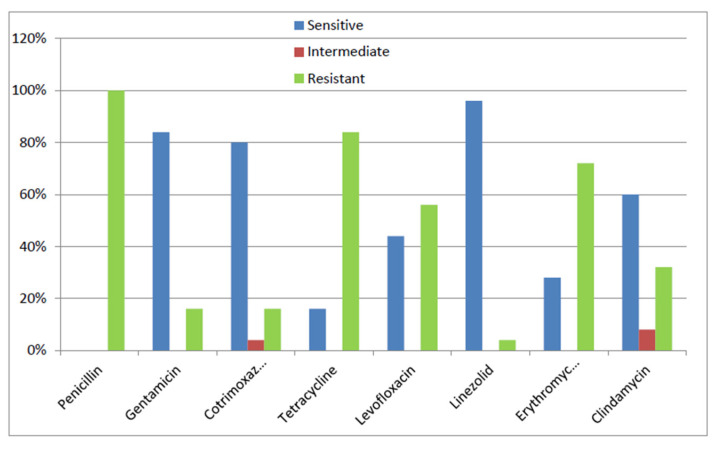
Antimicrobial susceptibility pattern of MSSA isolates.

On the other hand, MRSA isolates were least sensitive to penicillin and erythromycin. All MRSA isolates were resistant to penicillin, and only 28% were sensitive to erythromycin. All tested MSSA isolates were non-susceptible to penicillin. However, only 16% of MSSA tested isolates were sensitive to tetracycline. Regarding other tested antimicrobial agents, MRSA and MSSA isolates showed markedly variable sensitivity results (Figure 1 and Figure 2). In our study, MSSA isolates showed a statistically significant higher rate of resistance than MRSA isolates to both tetracycline (*p*-value 0.008) and levofloxacin (*p*-value 0.023).

Out of 50 MRSA isolates, 12 and 16 isolates presented constitutive and inducible clindamycin resistance, respectively. Out of 50 MSSA isolates, about 4 and 12 isolates presented constitutive and inducible clindamycin resistance, respectively. Using the Pearson chi-square test, no statistically significant difference was detected between MRSA and MSSA groups regarding the pattern of clindamycin resistance (*p*-value = 0.236).

### 3.2. Biofilm-Formation and Its Relation to Methicillin Resistance

About 87% of the isolates were biofilm-forming. Strong biofilm-formation was seen among 6 MRSA isolates and 2 MSSA isolates.

Moderate biofilm-formation was detected in about 15 MRSA isolates and 13 MSSA isolates. Finally, weak-biofilm formation was detected in 26 MRSA isolates and 25 MSSA isolates. On the other hand, 13% of the isolates were non-biofilm-forming, including three MRSA and ten MSSA isolates.

The correlation between biofilm-formation and methicillin susceptibility was determined using the Pearson chi-square test. MRSA isolates were significantly more capable of producing biofilm than MSSA isolates, as shown in Table 1 (*p*-value = 0.037).

### 3.3. Correlation between mazEF Toxin-Antitoxin Gene Expression and Methicillin Resistance

**Figure 3:**
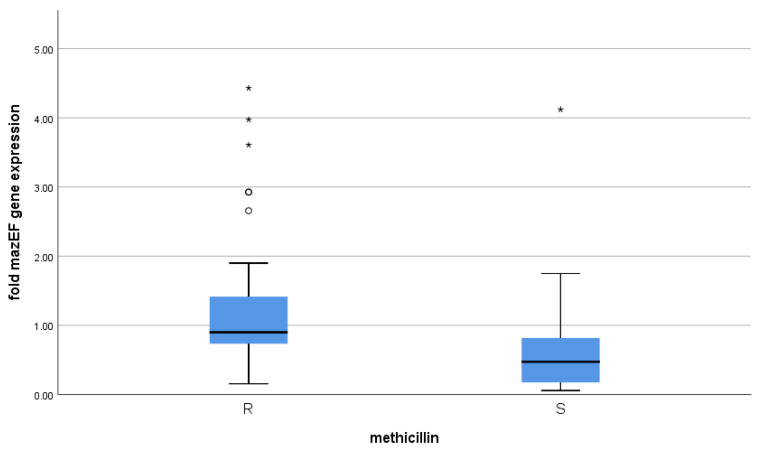
The *mazEF* expression in relation to methicillin susceptibility. On the boxplot, circles ° represent outliers and stars * represent more extreme values.

The *mazEF* expression values were highly variable and not normally distributed; therefore, we depended on the median and the inter-quartile range, not the mean, and utilized a non-parametric method (Independent-Samples Mann–Whitney U Test). Using the Mann–Whitney test, *mazEF* gene expression was significantly related to methicillin resistance (*p*-value < 0.001) (Table 2 and Figure 3).

### 3.4. Correlation between mazEF Toxin-Antitoxin Gene Expression and Biofilm-Formation

**Figure 4:**
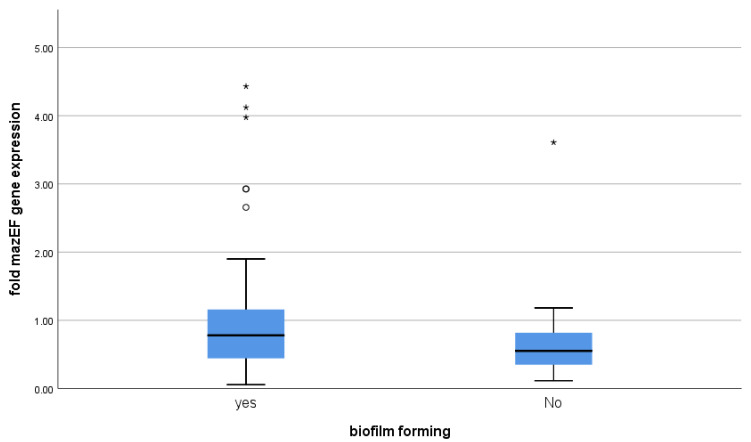
The *mazEF* expression in relation to biofilm-formation. On the boxplot, circles ° represent outliers and stars * represent more extreme values.

Using the Mann–Whitney test, *mazEF* gene expression was not linked to biofilm-formation (*p*-value = 0.136) (Table 3 and Figure 4).

## 4. Discussion

Deaths from infections caused by MRSA represent a major concern in hospitals. Antibiotic resistance in *S. aureus* has been observed against even the last line of therapy such as vancomycin, daptomycin, and linezolid [18]. In addition to developing resistance in their planktonic form, those bacteria are known to form a biofilm that enables them to evade antimicrobials and host defense mechanisms [18].

There are seven detected classes of TA systems. Under normal conditions, the antitoxin neutralizes the toxin to avoid the harmful toxin effects on the bacterial cell. In stress conditions, the labile antitoxin is destroyed, relieving the toxin which inhibits a vital cell process causing either persister formation or cell death [7,19]. In our study, we examined the expression level under normal conditions.

Our study showed that the best sensitivity results were observed with linezolid and gentamicin among MRSA or MSSA isolates. This agrees with other reports [20,21]. In our study, MRSA isolates were least sensitive to penicillin and erythromycin, while MSSA isolates were least sensitive to penicillin and tetracycline. On the contrary, better sensitivity results for erythromycin among MRSA isolates and for tetracycline among MSSA isolates have been obtained in other studies [20,21].

In this research, regarding inducible clindamycin resistance, it was observed with MRSA isolates. However, no statistically significant association was detected between methicillin resistance and the pattern of clindamycin resistance. This was similarly clarified in other studies [21,22]. Differences in *S. aureus* susceptibilities to different antimicrobials are caused mainly by geographical distribution, public health, infection control measures, and population awareness.

Bacteria within the biofilm are hardly affected by host immune defenses and antimicrobial agents, resulting in persistent destructive infectious diseases [18].

In this research, the biofilm-formation was reported in about 94% of MRSA isolates and 80% of MSSA isolates and was statistically significantly greater in MRSA isolates than MSSA (*p*-value = 0.037). Our results were in line with other recent studies [23,24].

The toxin of the *mazEF* system in *S. aureus*, mazF, induces growth arrest based on its endoribonuclease activity under stressful stimuli [25].

Concerning *mazEF* expression in normal conditions, our results showed that it was significantly linked to methicillin resistance (*p*-value < 0.001). Similarly, susceptibility among *S. aureus* isolates was improved upon deletion of the *mazF* gene, ensuring that *mazEF* expression is related to antibiotic resistance, according to a study by Ma et al. [25]. Similar results were observed by Schuster et al. [26]. Bacterial stasis caused by the mazF protein may account for resistance to antibiotics [27]. Again, this supports our results as *mazEF* expression among MRSA isolates was higher.

Concerning biofilm-forming isolates, we found no statistically significant difference in the expression of *mazEF* genes compared with non-biofilm-forming isolates (*p*-value = 0.136). In concordance with our findings, no significant relationship was detected between *mazEF* positive isolates and biofilm-formation among 150 *Escherichia coli* isolates [28]. Moreover, TA systems did not contribute to forming biofilms among *Streptococcus mutans* isolates. The authors declared that *mazF* and *relE* deletion has no effect on biofilm [29].

Apart from our results, a study found that biofilm-formation increased upon decreasing *mazF* expression. They concluded that *mazF* prevents biofilm-formation by disrupting the *ica* transcript [25]. Deletion of *mazF* gene increased *S. aureus* biofilm-formation [30]. The difference between our study and the previous two studies might be attributed to the study design where we studied *mazEF* expression in biofilm-forming versus non-biofilm-forming isolates rather than the same isolates before and after *mazEF* deletion.

In conclusion, our study reveals the aggravating resistance of *S. aureus,* especially MRSA isolates, to chemotherapeutic antimicrobials. Finding an alternative treatment strategy to chemotherapeutic antimicrobials is mandatory. The *mazEF* system is a new appealing antimicrobial target, and additional studies are required to validate its role in this prescriptive. Increasing the toxin mazF expression or preventing its neutralizing by the antitoxin mazE would eventually damage vital mRNA transcripts in a bacterial cell leading to its death. Finally, there is a need for more studies to declare the exact link between the *mazEF* system and biofilm-formation.

## Figures and Tables

**Table 1:** Biofilm-formation in relation to methicillin susceptibility.

	MRSA		MSSA		*p*-Value
	Count	%	Count	%	
Biofilm-forming isolates	47	94.0%	40	80.0%	0.037 *
Non-biofilm-forming isolates	3	6.0%	10	20.0%	

* Significant *p*-value ˂ 0.05.

**Table 2:** The *mazEF* expression in relation to methicillin susceptibility.

	Methicillin						
	R			S			*p* Value
	Median	1st Quartile	3rd Quartile	Median	1st Quartile	3rd Quartile	
Fold *mazEF* gene expression	0.90	0.73	1.42	0.47	0.17	0.82	<0.001 *

* Significant *p*-value ˂ 0.05.

**Table 3:** The *mazEF* expression in relation to biofilm-formation.

	Biofilm Forming						
	Yes			No			*p* Value
	Median	1st Quartile	3rd Quartile	Median	1st Quartile	3rd Quartile	
fold mazEF gene expression	0.78	0.42	1.18	0.55	0.35	0.82	0.136

## Data Availability

The data have been mentioned in the article.

## References

[1] Lee A.S., De Lencastre H., Garau J., Kluytmans J., Malhotra-Kumar S., Peschel A., Harbarth S. (2018). Methicillin-resistant *Staphylococcus aureus*. Nat. Rev. Dis. Prim..

[2] Pantosti A., Sanchini A., Monaco M. (2007). Mechanisms of antibiotic resistance in *Staphylococcus aureus*. Future Microbiol..

[3] Briggs T., Blunn G., Hislop S., Ramalhete R., Bagley C., McKenna D., Coathup M. (2017). Antimicrobial photodynamic therapy-a promising treatment for prosthetic joint infections. Lasers Med. Sci..

[4] O’Gara J.P. (2007). *Ica* and beyond: Biofilm mechanisms and regulation in *Staphylococcus epidermidis* and *Staphylococcus aureus*. FEMS Microbiol Lett..

[5] Page R., Peti W. (2016). Toxin-antitoxin systems in bacterial growth arrest and persistence. Nat. Chem. Biol..

[6] Fozo E., Makarova K.S., Shabalina S.A., Yutin N., Koonin E.V., Storz G. (2010). Abundance of type I toxin–antitoxin systems in bacteria: Searches for new candidates and discovery of novel families. Nucleic Acids Res..

[7] Wang X., Yao J., Sun Y.-C., Wood T.K. (2020). Type VII Toxin/Antitoxin Classification System for Antitoxins that Enzymatically Neutralize Toxins. Trends Microbiol..

[8] Aizenman E., Engelberg-Kulka H., Glaser G. (1996). An Escherichia coli chromosomal “addiction module” regulated by guanosine [corrected] 3′,5′-bispyrophosphate: A model for programmed bacterial cell death. Proc. Natl. Acad. Sci. USA.

[9] Schuster C.F., Bertram R. (2016). Toxin-antitoxin systems of *Staphylococcus aureus*. Toxins.

[10] Williams J.J., Hergenrother P.J. (2012). Artificial activation of toxin–antitoxin systems as an antibacterial strategy. Trends Microbiol..

[11] Clinical and Laboratory Standards Institute (2019). Zone Diameter And Minimal Inhibitory Concentration Interpretive Standards for S. aureus.

[12] Piechota M., Kot B., Frankowska-Maciejewska A., Grużewska A., Woźniak-Kosek A. (2018). Biofilm Formation by Methicillin-Resistant and Methicillin-Sensitive *Staphylococcus aureus* Strains from Hospitalized Patients in Poland. BioMed Res. Int..

[13] Cafiso V., Bertuccio T., Santagati M., Demelio V., Spina D., Nicoletti G., Stefani S. (2007). *agr*-Genotyping and transcriptional analysis of biofilm-producing *Staphylococcus aureus*. FEMS Immunol. Med. Microbiol..

[14] Williams J.J., Halvorsen E.M., Dwyer E.M., DiFazio R.M., Hergenrother P.J. (2011). Toxin-antitoxin (TA) systems are prevalent and transcribed in clinical isolates of Pseudomonas aeruginosa and methicillin-resistant *Staphylococcus aureus*. FEMS Microbiol. Lett..

[15] Monday S., Bohach G. (1999). Use of Multiplex PCR To Detect Classical and Newly Described Pyrogenic Toxin Genes in *Staphylo-coccal isolates*. J. Clin. Microbiol..

[16] Coskun U.S.S., Cicek A.C., Kilinc C., Guckan R., Dagcioglu Y., Demir O., Sandalli C. (2018). Effect of mazEF, higBA and relBE toxin-antitoxin systems on antibiotic resistance in *Pseudomonas aeruginosa* and *Staphylococcus isolates*. Malawi Med. J..

[17] Chan Y.H. (2003). Biostatistics 102: Quantitative data--parametric & non-parametric tests. Singap. Med. J..

[18] Craft K.M., Nguyen J.M., Berg L.J., Townsend S.D. (2019). Methicillin-resistant *Staphylococcus aureus* (MRSA): Antibiotic-resistance and the biofilm phenotype. Med. Chem. Comm..

[19] Harms A., Brodersen D.E., Mitarai N., Gerdes K. (2018). Toxins, Targets, and Triggers: An Overview of Toxin-Antitoxin Biology. Mol. Cell.

[20] Ahmed N.J. (2020). The Resistance of Staphylococcus Species to Different Antibiotics in Al-Kharj City. J. Pharm. Res. Int..

[21] Gurung R.R., Maharjan P., Chhetri G.G. (2020). Antibiotic resistance pattern of *Staphylococcus aureus* with reference to MRSA isolates from pediatric patients. Futur. Sci..

[22] Weiss L., Lansell A., Figueroa J., Suchdev P.S., Kirpalani A. (2020). Declining Prevalence of Methicillin-Resistant *Staphylococcus aureus* Septic Arthritis and Osteomyelitis in Children: Implications for Treatment. Antibiotics.

[23] Abu Haneen Z.S., Al-Hamadany W.S. (2019). Relation between biofilm formation ability and antibiotics resistance in *Staphylococcus aureus* from suppurative post-operation infections. Ann. Tro.p Med. Public Health.

[24] Kadkhoda H., Ghalavand Z., Nikmanesh B., Kodori M., Houri H., Maleki D.T., Bavandpour A.K., Eslami G. (2020). Characterization of biofilm formation and virulence factors of *Staphylococcus aureus* isolates from paediatric patients in Tehran, Iran. Iran J. Basic Med. Sci..

[25] Ma D., Mandell J.B., Donegan N.P., Cheung A.L., Ma W., Rothenberger S., Shanks R.M.Q., Richardson A.R., Urish K.L. (2019). The Toxin-Antitoxin *Maz*EF Drives *Staphylococcus aureus* Biofilm Formation, Antibiotic Tolerance, and Chronic Infection. mBio.

[26] Schuster C.F., Mechler L., Nolle N., Krismer B., Zelder M.-E., Götz F., Bertram R. (2015). The MazEF Toxin-Antitoxin System Alters the β-Lactam Susceptibility of *Staphylococcus aureus*. PLoS ONE.

[27] Fu Z., Tamber S., Memmi G., Donegan N.P., Cheung A.L. (2009). Overexpression of MazF_Sa_ in *Staphylococcus aureus* Induces Bacterio-stasis by Selectively Targeting mRNAs for Cleavage. J. Bacteriol..

[28] Karimi S., Ghafourian S., Kalani M.T., Jalilian F.A., Hemati S., Sadeghifard N. (2014). Association Between Toxin-Antitoxin Systems and Biofilm Formation. Jundishapur J. Microbiol..

[29] Lemos J.A., Brown T.A., Abranches J., Jr., Burne R.A. (2005). Characteristics of Streptococcus mutans strains lacking the MazEF and RelBE toxin-antitoxin modules. FEMS Microbiol. Lett..

[30] Kato F., Yabuno Y., Yamaguchi Y., Sugai M., Inouye M. (2017). Deletion of *mazF* increases *Staphylococcus aureus* biofilm formation in an *ica*-dependent manner. Pathog. Dis..

